# Polygenic risk scores: An overview from bench to bedside for personalised medicine

**DOI:** 10.3389/fgene.2022.1000667

**Published:** 2022-11-11

**Authors:** Benjamin Cross, Richard Turner, Munir Pirmohamed

**Affiliations:** The Wolfson Centre for Personalised Medicine, Institute of Systems, Molecular and Integrative Biology, Faculty of Health & Life Sciences, University of Liverpool, Liverpool, United Kingdom

**Keywords:** metabolic disease, polygenic risk score, personalised medicine, pharmacogenomics, genetic risk score, cardiovascule disease, neuropsychiatric disorders, cancer

## Abstract

Since the first polygenic risk score (PRS) in 2007, research in this area has progressed significantly. The increasing number of SNPs that have been identified by large scale GWAS analyses has fuelled the development of a myriad of PRSs for a wide variety of diseases and, more recently, to PRSs that potentially identify differential response to specific drugs. PRSs constitute a composite genomic biomarker and potential applications for PRSs in clinical practice encompass risk prediction and disease screening, early diagnosis, prognostication, and drug stratification to improve efficacy or reduce adverse drug reactions. Nevertheless, to our knowledge, no PRSs have yet been adopted into routine clinical practice. Beyond the technical considerations of PRS development, the major challenges that face PRSs include demonstrating clinical utility and circumnavigating the implementation of novel genomic technologies at scale into stretched healthcare systems. In this review, we discuss progress in developing disease susceptibility PRSs across multiple medical specialties, development of pharmacogenomic PRSs, and future directions for the field.

## 1 Introduction

Predicting risk of disease and response to treatment are essential components of clinical medicine, and often direct the subsequent management strategy. Currently in clinical medicine, the majority of prediction is based on basic clinical factors such as age, sex and family history, alongside biochemical biomarkers and radiological imaging.

Since the completion of the Human Genome Project, there has been significant growth in genetic research with the mass undertaking of genome-wide association studies (GWAS) and, more recently, rare variant and gene-based testing leveraging whole exome or genome sequencing (WES/WGS) endeavours. Initial expectations that genomics would rapidly transform and personalise medicine have been largely unmet, attributable to a range of factors including greater disease genetic complexity than initially expected and challenges identifying the causal gene(s) within associated loci. Nevertheless, genetic testing is slowly being introduced in a number of areas including identification of mutations for rare diseases and identification of driver mutations in tumours to enable targeted therapies. In addition, single gene screening to guide drug prescribing include HLA-B*57:01 testing with abacavir (Mallal et al., 2008) and HLA-B*15:02 screening in the Han Chinese population prior to starting carbamazepine (Ferrell and McLeod, 2008). Notwithstanding, efforts over the last few years to stratify treatment and screening have started to demonstrate promise with polygenic risk scores (PRSs).

Initially, the development of PRSs focussed on predicting disease risk leveraging genome-wide significant single nucleotide polymorphisms (SNPs) with *p*-values typically <5 × 10^−8^, which constrained predictive risk scores due to the limited number of SNPs included (Evans et al., 2009). As the sample size of GWAS studies grew, evidence suggested many common adult-onset diseases are mediated by numerous common (minor allele frequency (MAF) > 5%) and low frequency (MAF 0.5< x <5%) genetic variants. These variants, individually, have little contribution, but together form a complex network that can make a patient more or less susceptible to disease (Torkamani et al., 2018; Lambert et al., 2019). However, with the advent of large sequencing efforts, it is also important to consider the role of rare variants in predisposing to common disease (Schork et al., 2009; Bomba et al., 2017). For example, a study of 36 individuals with autism in the Faroe islands showed that rare and common contributed to the disease (Leblond et al., 2019). A more recent report in 24,248 schizophrenia cases showed that ultra-rare coding variants in 10 genes highly expressed in the central nervous system conferred a significant risk of schizophrenia with odds ratios ranging from 3 to 50 (Singh et al., 2022).

To our knowledge, there are currently no germline PRSs in clinical use; however, multiple PRSs are being assessed in clinical trials across a range of disciplines. Here, we review the utility of PRSs in the context of risk prediction, guiding pharmacotherapy, touch on PRSs in diagnosis, and finally, we explore the potential future of PRSs within modern healthcare. We initially focus on PRSs in major disease areas, where the majority of work has been undertaken, and then on work on PRSs in prediction of drug efficacy and safety. To some extent, this is an artificial distinction since work in disease PRSs may also stratify populations to enable the use of treatments at an earlier stage in some of the genomically-defined subgroups.

In order to prepare this narrative review, we have used generic search terms such as polygenic scores, specific disease terms, disease risk, disease stratification, pharmacogenomics, drug efficacy and drug safety, ethnicity and ancestry in large databases such as PubMed to identify the relevant papers. In addition, we have also searched the cited references contained within the retrieved articles.

## 2 Utility in disease pathogenesis and screening

### 2.1 Cardiovascular disease

The Framingham study led to the Framingham risk score including age, sex, LDL cholesterol, HDL cholesterol, hypertension, diabetes and smoking, which has influenced cardiovascular disease (CVD) risk assessment (Wilson et al., 1998). Although the Framingham risk score has proven utility, it relies on a snapshot of a patient’s risk factors, which vary with time. In contrast, the germline genome is static and offers improved capture of lifelong CVD risk exposure (Voight et al., 2012; Zannad et al., 2012; Knowles and Ashley, 2018).

Globally, CVD accounts for around 31% of all mortality. The World Health Organisation (WHO) estimates that 75% of premature cardiovascular (CV) death is preventable. The INTERHEART study demonstrated that lifestyle changes can be protective against future events, underpinning the importance of primary prevention (Yusuf et al., 2004; Stewart et al., 2017). Coronary artery disease (CAD) GWAS studies have identified over 160 genome-wide significant loci involving more than 200 SNPs not in linkage disequilibrium (Mega et al., 2015; Vaara et al., 2016; Morieri et al., 2018; Rincon et al., 2019). Incorporation of increasing numbers of SNPs in CAD PRS models has improved predictability in addition to traditional risk factors, albeit with only modest increases in area under the receiver operator curve (AUC) (Natarajan et al., 2017; Morieri et al., 2018). For example, a PRS including 6,630,150 polymorphisms recently demonstrated an AUC >0.8 for prediction of CAD and enabled risk stratification of those at much higher risk of CAD with odds ratios (ORs) of 3, 4 and 5 for those in the top 8, 2.3 and 0.5% of the PRS, respectively. (Khera et al., 2018). Thus, these high-risk patient groups could be targeted to receive preventative strategies. Utilising this same PRS, a recent study assessed prediction of incident CHD events compared with risk prediction using a guideline recommended clinical risk equation (10-year risk based on the 2013 ACC/AHA pooled cohort equations). Similar ORs for CAD development were found in their study cohorts; however the PRS offered minimal improvement over clinical modelling in reclassification as high/low risk in less than 10%. Furthermore, of those who went on to develop CAD, 79–80% of those who had been reclassified were incorrect (Mosley et al., 2020).

PRSs can provide prognostic information independent of classical risk factors, including family history, and guide primary prevention (Assimes and Roberts, 2016; Morieri et al., 2018; Roberts, 2018). This is exemplified by a recent prospective study by Khera et al. (2016) that demonstrated the relative risk of coronary events was 91% higher in those deemed at high genetic risk than those at low risk. Those at high genetic risk with modified lifestyle had a near 50% decrease in risk compared to those without. Various measures of fitness and physical activity demonstrated inverse associations with future CVD events and all-cause death. This had a graded effect with genetic risk models for CVD and atrial fibrillation (AF) (Tikkanen et al., 2018).

The clinical utility of targeted testing following a primary acute coronary syndrome (ACS) event to guide secondary preventative measures has potential clinical application. Evidence for PRSs in the setting of recurrent coronary artery events is limited to four studies to date, which show mixed results. There is suggestion that CAD recurrence or cardiac death are distinct phenotypes from the initial CV event, with likely additional distinct genetic variants. One study investigated 22 variants derived from a first event prediction cohort and identified only two of those variants to be significant predictors of recurrent cardiovascular events (Wauters et al., 2013). Vaara et al. (2016) developed three novel PRSs using the CARDIOGRAMplusC4D cohort containing 32, 47 and 153 SNPs. The 47 SNP model demonstrated a significant association with recurrent ACS events, although there was no increase in AUC compared with or in addition to clinical predictors. Rincon et al. (2019) demonstrated positive results with a smaller patient cohort of around 500 patients and a PRS featuring 11 SNPs. Those in the highest risk tertile had a significant hazard ratio of 3 for recurrent events. Recurrent events in other risk categories were non-significant. A modest increase in AUC when combined with clinical models was observed (0.78 and 0.83 respectively). Finally, Mega et al. (2015) evaluated a 27 SNP PRS in three primary prevention populations and two secondary prevention trial populations. Using meta-analysis of secondary prevention results, they demonstrated significant differences in hazard ratio between risk tertiles.

Overall, despite heritability estimates for atherosclerosis ranging from 30 to 60% (Lusis, 2012), CAD PRSs, like the PRSs of many chronic conditions, currently only account for ∼4% of observed variance (Khera et al., 2018). Therefore, it is anticipated that the clinical value for utilising PRSs will be mainly experienced by those at the extremes of the genetic risk distribution and/or those on the threshold cusps for clinical decisions based on conventional clinical assessment (e.g. individuals near the risk threshold for being recommended primary prevention statin therapy). Nevertheless, the high prevalence of CAD and greater influence of genetic risk upon cardiovascular events at younger ages suggest that the absolute number who could benefit from utilising CAD PRS scoring could be sizeable. Thus, a pilot study in the NHS has been initiated that will integrate a CAD PRS with standard QRisk-based scoring to provide refined risk predictions in 1,000 45–64 year olds that can be used to guide primary prevention clinical decision-making (such as starting statin therapy) (Health Education England, 2021; Devlin, 2022).

AF is the most common cardiac arrhythmia and is associated with thromboembolic stroke, heart failure and death. The oftentimes paroxysmal nature of AF can make diagnosis challenging and treatment with anticoagulation is not without significant risk. Everett et al. (2013) sought to develop an improved clinical risk score and assess additional benefit of a novel PRS in a large cohort (>20,000) of female participants. While addition of the PRS improved the risk score AUC, it failed to significantly aid the classification of patients into 10-year risk categories estimating the development of AF. Contrary to these findings, a 12 SNP PRS not only identified differences in AF risk between quintiles, but compared to the CHADS_2_ clinical score, the PRS prompted some risk reclassification and increased risk prediction of ischaemic stroke (Tada et al., 2014). Muse et al. (2018) demonstrated a greater than 3-fold risk increase for AF between the highest and lowest quintile with their PRS model. Further, larger PRSs, utilising 6,730,541 polymorphisms identified 6.1% of the population studied to have a three-fold increase in AF risk and was able to identify those with an increasing OR of 3, 4 and 5 for patients within the top 6.1, 1.5 and 0.7% of genetic risk scores, respectively (Khera et al., 2018). The clinical utility of these PRSs may lie in targeting extended ECG monitoring in patients at increased risk (Khera et al., 2018; Muse et al., 2018), as well as aiding investigation in cryptogenic stroke (Muse et al., 2018).

### 2.2 Long QT

QT prolongation is associated with an increased risk of Torsades de Pointes (TdP) and sudden cardiac death, and is an important adverse reaction for many medications. QT prolongation remains a leading cause for drugs being withdrawn from the healthcare market. The QT interval is heritable, with estimates of between 30 and 40 percent of variance due to cumulative genetic factors (Rosenberg et al., 2017). The largest data from European ancestry individuals has been provided by the QT Interval–International GWAS Consortium (>70,000 individuals) (Arking et al., 2014) and, in individuals of African descent, the CARe-COGENT consortium (>12,000 individuals) (Smith et al., 2012). In 2017, from emerging genetic data linked with prolonged QT syndromes, a large cohort PRS explained a significantly increased amount of variation in the QT interval, compared with a non-genetic model in those of European descent, but not individuals of African American descent (Rosenberg et al., 2017). Furthermore, an initial pilot study demonstrated a PRS was associated with QT prolongation and the PRS also predicted the development of drug-induced TdP (Strauss et al., 2017). Building on this, a recent publication assessed prediction of cardiac electrical response to sodium-channel blockade as well as Brugada syndrome. Here, it was demonstrated that genetic factors underlie variation in electrical response to sodium channel blockers to a clinically significant level. Combining a PRS, electrocardiogram (ECG) and family history, one could predict the development of drug-induced Type I Brugada syndrome (Tadros et al., 2019). The clinical utility of this score may allow a reduction in investigation-related adverse events, such as life-threatening arrhythmias with the use of diagnostic ajmaline (around 1.8%) (Conte et al., 2013), as well as reducing toxicity associated with sodium channel blockade through pre-drug screening or risk assessment based follow up with ECGs (Tadros et al., 2019).

### 2.3 Cancer

Breast cancer is the most common cancer in women in Western countries and, although *BRCA1* and *BRCA2* variants are well documented risk factors for disease development, they only account for a fraction of breast cancer cases (Mavaddat et al., 2019). An in-depth review of the use of PRSs exclusively in breast cancer is beyond the scope of this review, but can be located in a contemporary review by Yanes et al. (2020). Nevertheless, the biobanking initiatives from the Breast Cancer Association Consortium and others have enabled development of multiple PRSs based on large scale, robust data. A recent study constructed a PRS using 5,218 SNPs from a previous GWAS with linkage disequilibrium analysis based on Europeans in the 1000 Genomes Project, before validating the results in United Kingdom Biobank. This PRS was able to identify women at increased risk for breast cancer with ORs of 3, 4 and 5 corresponding to women within the upper 1.5, 0.3 and 0.1% of genetic risk scores, respectively. Women in the top 0.1% of genetic risk scores had a disease prevalence of 19%, compared with 4.2% in the remaining 99.9% of the cohort. Breast cancer screening in asymptomatic middle-aged women remains controversial due to the low incidence of disease in this cohort and high false positive rate. This PRS demonstrates a possible method to provide more targeted screening (Khera et al., 2018) rather than basing breast cancer screening on age alone (Pashayan et al., 2011; Hall and Easton, 2013). Similarly reported that small groups of patients with a high risk of disease can be identified with risk scores in the top 1% of the population studied (Mavaddat et al., 2015; Mavaddat et al., 2019). In the United Kingdom, women become eligible for the breast cancer screening programme at age 47, when the average absolute risk of breast cancer over 10 years is 2.6%. However importantly, Mavaddat et al. (2019) highlighted that 19% of those in the highest PRS risk group will reach this 10-year risk threshold on average at age 40, whereas women with very low genetic risk may never surpass this threshold. NICE guidance advocates the use of chemoprevention for 5 years in post-menopausal women who are at high risk of breast cancer and for chemoprevention consideration for those at moderate risk. Identification of those at risk can be refined by use of a PRS alongside clinical risk evaluation, although prospective trials to investigate this strategy further are required (Garcia-Closas et al., 2014).

Genetic risk scoring in prostate cancer first started in 2008 when a five SNP PRS was shown to account for 46% of all prostate cancer cases when combined with family history in a cohort of Swedish men (Zheng et al., 2008). The validity of this score was confirmed in a United States population (Salinas et al., 2009) and, subsequently, multiple additional SNPs have been identified that provide incremental improvement in predicting biopsy positive prostate cancer, compared with family history alone (Kader et al., 2012; Sun et al., 2013; Liss et al., 2015). The genetic score acts independently of reported family history, indicating additional benefit (Amin Al Olama et al., 2016). This PRS benefit could be realised through stratification of patients based on their genetic risk score to guide screening. One particular model, PGS-33, demonstrated an increase in prostate cancer detection from lower to higher PRS quartiles (Liss et al., 2015), and data from the PRACTICAL study illustrated a significant difference in risk between the upper 1% and lowest 1% of the population based on their genetic risk score (OR 4.2 and 0.14, respectively) (Amin Al Olama et al., 2016). Aly et al. (2011) explored the potential clinical utility of PRSs in relation to healthcare resources. They compared a nongenetic model (based on age, prostate specific antigen (PSA), free-to-total PSA, and family history) with a genetic model (including 35 SNPs) and identified that 480 biopsies (22.7%) could have been avoided using the PRS as a decision tool, although a diagnosis of prostate cancer would have been missed in 3% of patients that were classed as having aggressive disease. Many of the prostate cancer PRS studies do not contain replication cohorts likely due to sample size issues.

### 2.4 Neuropsychiatric diseases

Neuropsychiatric PRS development is a growing field largely due to the polygenic nature of all mental health disorders but also due to the debilitating effects on patients, population burden of disease and the effectiveness of the medications (covered later). Here, we focus on schizophrenia and major depressive disorder.

### 2.5 Schizophrenia

PRSs for schizophrenia currently explain the greatest proportion of phenotypic variance compared with PRS for other neuropsychiatric conditions (Binder, 2019). Additionally, the predictive power of schizophrenia PRSs exceeds that of other common diseases (Landi et al., 2021). One initiative identified 108 novel loci associated with schizophrenia in a large case-control study. A PRS from this data demonstrated that the odds of developing schizophrenia were 7.8–20.3 fold greater in those in the top decile compared to those in the bottom decile of the PRS, although the specific odds varied depending on the cohort tested (Schizophrenia Working Group of the Psychiatric Genomics, 2014). Utilising the same PRS, another working group demonstrated a correlation between PRS scores and admission frequency (Meier et al., 2016). However, psychiatric diagnosis in clinical trial settings is often highly ascertained through lengthy interview processes with “clean” patients with no previous mental health disorders. Often in real-world settings, schizophrenia diagnoses are not so clear cut and are often confounded by concomitant mental health disorders. A further PRS was derived from the same GWAS data and tested to ascertain how a schizophrenia PRS could function in real world healthcare settings. Results were replicated in retrospective cohorts from four different healthcare systems. Importantly, the PRS was robustly associated with schizophrenia and the ORs for schizophrenia remained elevated, albeit more modestly, when comparing the top to the bottom PRS deciles (ORs 2.3–4.6) (Zheutlin et al., 2019). A smaller but multi-ethnic study in a United Kingdom population demonstrated that a PRS showed good discrimination between patients with first episode psychosis and controls in European ancestry individuals (9.4% of variance accounted for), but more modest discrimination in those of African ancestry (1.1% of variance explained). In European ancestry patients, the PRS distinguished between those that developed a diagnosis of schizophrenia compared to those that developed other psychotic disorders, suggesting that a PRS may aid diagnosis in patients with a first episode psychosis. This study also highlights the importance of conducting genetic research in different ancestral groups to improve PRS generalisability and avoid introducing additional healthcare inequalities (Vassos et al., 2017).

The utility of PRSs in screening for schizophrenia is likely limited by the low disease prevalence. The top 10% genetic risk stratum carries an estimated average three-fold increased risk of developing schizophrenia. However, as the population prevalence of schizophrenia is just 1%, only 3% of this “high risk” stratum will develop schizophrenia. Even in the top 1% genetic risk stratum, only 6% will develop schizophrenia (Murray et al., 2021). Thus, additional risk factors will need to be integrated with PRSs if population schizophrenia risk screening is to become more viable.

### 2.6 Depression

Heritability of major depression in twin studies has been demonstrated to be between 30 and 40% with SNP-based heritability measures of 9–37% (Sullivan et al., 2012; Musliner et al., 2019). The current understanding of gene heritability in major depressive disorder (MDD) in PRSs explains only around 3.6% of the variance (Ni et al., 2021).

One PRS score in a Danish case-cohort study demonstrated a 30% increased risk of receiving a diagnosis of depression before the age of 31 for each standard deviation increase in polygenic liability (Musliner et al., 2019). In this same discovery Danish cohort (iPSYCH 2012), a study investigating the effects of PRS, socio-economic status and parental psychiatric history found that the absolute risk of depression by 30 years of age differed notably, depending on an individual’s combination of these risk factors. Importantly, this risk of depression was almost 24% among women in the top 2% of the PRS distribution with a parental history of psychiatric disorders, illustrating the potential of multivariable risk score systems (Agerbo et al., 2021).

Most MDD PRS research has focussed on early onset or initial presentation. However, there is a significant prevalence of depressive symptoms and depression in older adults (Kessler et al., 2003; Zivin et al., 2010). One study utilising 11 SNPS in an older patient cohort found that a one standard deviation increase in PRS was associated with an approximate OR of 1.08 increase in mean depressive symptom score, equating to approximately 18% of the effect size of being female. They also found a (non-significant) trend that as the PRS standard deviation increased, participants were more likely to consistently report high levels of depressive symptoms over time (Levine et al., 2014).

A study by Peyrot et al. (2014) assessed genetic × environment interactions and found that PRS effects are modulated by environmental factors. In particular, they demonstrated that the strength of the association between PRS and risk of depression increased in the presence of childhood trauma.

Unhealthy lifestyle and high genetic risk burden based on a depression PRS was associated with a two-fold increase in risk of depression compared with a healthy lifestyle and low PRS-determined genetic risk, although no formal statistical interaction was observed. The beneficial impact of healthy lifestyle was reported across all risk groups suggesting genetic predisposition could be counteracted by environmental factors (Cao et al., 2021).

In contrast to schizophrenia; the overall population risk of major depression is relatively high (15%). Therefore, it is estimated that 30% of those within the top 1% of the PRS distribution will develop depression (Murray et al., 2021), potentially making identification of at-risk individuals more tractable for targeted research and interventions.

A small case-control study utilising adult GWAS data extrapolated to adolescent cohorts, generated a PRS able to explain 7.9% of observed variance itself and, when combined with history of childhood abuse, explained 17.9% of the variance of future depression prediction. Similarly, they demonstrated the PRS in isolation explained 7.7% of the variance, and the additive model explained 13.5% of the variance of depression severity in adolescents (Halldorsdottir et al., 2019). They however did not report the variance explained by effects of childhood abuse alone for comparison. Nonetheless this does support PRS use in depression prediction in combination with clinical factors. Furthermore; a study using GWAS data from an adult Caucasian population developed a PRS to assess a Mexican youth cohort for development of depression. They too found a significant prediction of depression in this youth cohort; however, again effect sizes were small and the AUC of PRS models were modest (0.55–0.58) (Rabinowitz et al., 2020). Both studies demonstrate the potential of PRS to identify risk groups that could be targeted with preventative strategies, although the predictive capability of the models must increase.

PRSs can also provide insights into disease pathogenesis. One recent study generated a functionally informed PRS based on subtle transcriptomic shifts in gene expression towards a depression profile seen in key parts of the corticolimbic circuit. Interestingly, this PRS was associated with widespread reductions in neural response to neutral faces in women which, in turn, was associated with increased self-reported anhedonia. To corroborate this mechanistic PRS, the authors also studied a more traditional depression (disease) susceptibility PRS using variants from large scale case control GWAS literature data and found a similar association with blunted reactivity to neutral faces in women (Mareckova et al., 2020).

Two important considerations of PRS development in psychiatry are phenotypic heterogeneity and pleiotropy. First, it is inevitable that treated patients will show inter-individual variability in response, and therefore will display different symptom severity or a different constellation of symptoms after drug treatment. This phenotypic heterogeneity particularly after treatment has to be taken into account as it can impact endpoint definitions that, in turn, could impact PRS-based analyses (Santoro et al., 2018). Second, pleiotropy, the genetic effect of the same locus on multiple traits, can take the form of biological pleiotropy, where the locus contributes to multiple phenotypes, or mediated pleiotropy, where the locus increases the liability to a second disorder that occurs as a consequence of schizophrenia or its treatment (Zheutlin et al., 2019). A study of 106,160 patients not only showed a strong association with schizophrenia but also with anxiety, mood, substance use, personality disorders and suicidal behaviour (Zheutlin et al., 2019). Similarly, in a study in the United States Veterans Affairs Health Care System in patients with schizophrenia, bipolar disorder and depression, higher PRSs were associated with higher likelihood of mental and physical health diagnoses, although the effect size was lower in African ancestry individuals than in those of European ancestry (Bigdeli et al., 2022). A phenome-wide analysis of UK Biobank participants has shown that highly pleiotropic variants corresponded to ubiquitously expressed genes important for extracellular matrix, regulation of cell growth and signalling pathways (Shikov et al., 2020).

### 2.7 Neurodegenerative disease

Alzheimer’s disease (AD) is the most common form of dementia worldwide and constitutes one of the largest public health issues globally, given aging populations. The strongest known genetic link with AD is *APOE* ε4, although modern GWAS data have identified multiple distinct genetic loci. There have been many attempts to produce PRSs for AD with mixed results (Stocker et al., 2018). The *APOE* ε4 allelic risk is well documented; two copies of the allele result in increased AD risk as well as reduced age of onset, although the strength of this effect varies across different populations (Liu et al., 2013). PRS development, excluding *APOE* ε4, have demonstrated worse performance of disease prediction in healthy individuals than *APOE* ε4 status alone. However, those that incorporated *APOE* ε4 showed increased diagnostic accuracy compared with APOE ε4 alone (Stocker et al., 2018). While there is currently no clinical utility of genetic risk scores in AD, PRSs could potentially guide development of future treatment stratification and trial patient recruitment strategies to improve drug development.

Parkinson’s disease is an age-dependent neurodegenerative condition; it has been reported that, as the numbers of associated SNPs carried by individuals increases, disease onset occurs at an earlier age (Escott-Price et al., 2015; Nalls et al., 2015) and motor function and cognition declines more rapidly (Paul et al., 2018).

### 2.8 Metabolic disorders

Obesity is a major public health issue with a significant health-economic burden and has been shown to have a notable polygenic component (Yang et al., 2007; Belsky et al., 2013). Of note, obesity PRSs have been unable to accurately discriminate between those at high and low obesity risk, likely influenced by the strong environmental influence on the development of obesity. One model demonstrated that a PRS for obesity outperformed the monogenic risk loci with the strongest associations to date (Belsky et al., 2013), *FTO* and *MC4R*, which together predict around 0.59% of obesity variation (Loos and Janssens, 2017). Although an improvement, the AUC for this PRS was only slightly better than chance by itself (0.574) and after being combined with a socioeconomic score (0.586). The PRS also failed to predict BMI for African-American patients, highlighting the complexity of PRS development and lack of generalisability in an ethnically diverse population (Belsky et al., 2013). Current prediction models have improved with the addition of non-genetic obesity risk factors such as education, employment, diet, medication, smoking, and physical activity alongside the PRS, which collectively increase the AUC to 0.69 (Sandholt et al., 2010). In keeping with other conditions, obesity PRSs appear more informative for those at the extremes of the genetic risk distribution (Peterson et al., 2011).

Measurement of islet autoantibodies in children can indicate active type 1 diabetes mellitus (T1DM) years before clinical diagnosis. However, they are often difficult to obtain, are expensive, and have reduced sensitivity and specificity in adult cohorts. As T1DM is highly heritable with its twin concordance rate of around 70%, numerous PRSs have been developed that demonstrate AUCs of >0.8 (Winkler et al., 2014; Oram et al., 2016; Sharp et al., 2019). Although both *HLA-DR3* and *DR4-DQ8* are known to increase the risk of T1DM, recent studies have demonstrated increased risk stratification with additional SNPs in PRSs (Bonifacio et al., 2018). There is hope that early detection before clinical symptoms could lead to primary prevention trials and novel preventative treatments.

With rising levels of obesity, it is becoming increasingly difficult to distinguish type 2 diabetes mellitus (T2DM) from T1DM in young patients. Importantly, a T1DM PRS has been shown to be highly discriminative between T1DM and T2DM (Oram et al., 2016). Furthermore, this same T1DM PRS has been used in patients with T2DM in conjunction with GAD65 autoantibodies to detect those that were more likely to rapidly progress to needing insulin therapy (Grubb et al., 2019).

Both metformin and lifestyle intervention have been shown to prevent progression to T2DM, which in turn is a key clinical risk factor for cardiac and renal disease globally (Knowler et al., 2002). A T2DM PRS identified those within the top 3.5% of genetic risk scores to have a greater than three-fold average risk of having T2DM compared to the rest of the cohort; such an approach could prioritise costly preventative strategies and reduce those exposed to adverse drug reactions from prophylactic metformin (Khera et al., 2018). Furthermore, PRS studies have identified that risk scores add predictive value over traditional risk factors in T2DM, although discriminatory gains are modest (Vaxillaire et al., 2014; Lall et al., 2017).

### 2.9 Venous thromboembolism

Venous thromboembolism (VTE) is a multifactorial disease with complex interplay between acquired and genetic factors, affecting around 0.2% of the population in America and Europe annually. An estimated 60% of VTE risk is heritable, but currently only two established variants within the clotting cascade are used in clinical practice: Factor V Leiden (FVL), and the gain-of-function 20210G>A variant in prothrombin (Soria et al., 2014). However, the clinical utility of this genetic testing is controversial and limited (Stevens et al., 2016). Multiple additional common low-penetrance loci linked to unexpected genes have been identified in recent GWAS analyses (Morange and Tregouet, 2011; Sabater-Lleal et al., 2012; Tang et al., 2013). In predicting a first VTE episode, de Haan et al. (2012) derived a PRS from five highly significant SNPs to improve predictions when used in combination with clinical risk factors, compared to clinical risk factors alone. However, their work also demonstrated that a risk score combining 31 individual SNPs provided no significant added prognostic benefit over the five SNP model. Recent work supports these findings and has identified further SNPs that improve VTE prediction, but also suggest that low frequency variants with lower ORs add little to VTE prediction (Soria et al., 2014). When examining recurrent VTE episodes, patients with multiple (two or more) SNPs associated with VTE had a substantially higher risk of recurrence; however, the proportion of patients with multiple SNPs was limited to around 4% (van Hylckama Vlieg et al., 2008). Further issues arise when examining genetic risk of VTE in breast cancer patients. Patients in the upper 5% of genetic risk scores had a similar risk of VTE recurrence to family history and FVL status, although 93% of those in the upper 5% were FVL carriers suggesting that routine FVL testing alone may be sufficient (Brand et al., 2016).

### 2.10 Utility in adverse drug reactions and drug efficacy

#### 2.10.1 Statins

Statins are widely used group of drugs which lower LDL cholesterol and have been shown to be efficacious for both primary and secondary prevention of atherosclerotic arterial disease. Although measurement of cholesterol and/or clinical risk scores are used to determine when patients should be prescribed a statin, research in this area has attempted to identify whether PRSs may add value over and above clinical risk categorization with respect to the effect of statins in preventing cardiac events. Mega et al. (2015) demonstrated the potential of PRSs to categorise patients into different risk tertiles for coronary heart disease events, as described earlier. However, they also demonstrated different levels of statin efficacy across the risk groups. Most notably, from primary prevention trial data (JUPITER and ASCOT), they demonstrated an approximate three-fold difference in relative risk reduction between highest and lowest genetic risk tertiles. This translates to a decrease in the number needed to treat (NNT) with statins to prevent one coronary heart disease event over 10 years between the lowest and highest genetic risk tertile groups (low genetic risk NNT: Jupiter = 66, ASCOT = 57 and high genetic risk NNT: Jupiter = 25, ASCOT = 20). A further study analysed patients from the WOSCOPS primary prevention trial and developed a larger 57 variant PRS to further investigate the effects of statins. They coupled the outcome data with observational study data to explain phenotypic effects on coronary disease plaques and burden. They demonstrated a NNT of 13 in those carrying the highest genetic burden compared to a NNT of 38 in the rest of the study population, supporting the results seen by Mega et al. This effect also correlated with coronary artery calcification and plaque burden data, showing for every standard deviation increase in PRS, the coronary artery calcification and plaque burden correspondingly increased (Natarajan et al., 2017). Contrary to this evidence, however, one PRS demonstrated no significant difference between patient PRS tertiles and serum LDL levels after 6 months of statin therapy (Rincon et al., 2019). To our knowledge, there have yet to be any completed prospective studies utilising genetics on patient selection for statins in either primary or secondary prevention strategies. However, the addition of a PRS in a two-stage screening strategy for primary CVD prevention has been proposed and NHS pilot is underway (Health Education England, 2021; Devlin, 2022). The addition of the PRS could help reclassify and identify those at intermediate risk into higher risk categories who may then benefit from statin therapy (Tikkanen et al., 2013).

#### 2.10.2 Clopidogrel

Clopidogrel is prescribed for the prevention of atherothrombotic events in patients with coronary artery disease (Valgimigli et al., 2017) and as secondary prevention in stroke (The National Institute for Health and Care Excellence (NICE), 2020). Growing interest in interindividual patient variability in response to clopidogrel therapy has led to evidence indicating that a relatively high proportion of variability seen in response to clopidogrel is attributable to genetic variation (Shuldiner et al., 2009). A recent study has attempted to model this genetic variation in a PRS format. They used a database of 3,391 European ancestry patients on clopidogrel and assessed platelet reactivity *via* a variety of platelet function assays which were standardised to allow comparison. Clinical endpoints of cardiovascular events were then assessed in 2,134 of these patients. A PRS was developed that incorporated CYP2C19*2 and five SNPs that were significantly associated with platelet reactivity in the study. They observed that with an increasing number of risk alleles, patient’s platelet reactivity increased and so did their risk of cardiovascular events. Patients with eight or more alleles were more likely to experience cardiovascular events than those with six alleles (OR = 1.78 (CI 1.14–2.76, *p* = 0.01). However, the six SNPs included only accounted for 3.5% of the variation in platelet function (Lewis et al., 2020). Importantly after adjustment for CYP2C19*2, the remaining SNPs were non-significant for composite CV endpoints (Lewis et al., 2020). In another study, no other SNPs were statistically significant for platelet aggregation after CYP2C19*2 adjustment. However multiple regression analysis in that study population showed that a combination of CYP2C19*2, rs2254638, and rs2487032 could explain 28.2% of antiplatelet response (10.9%, 14.8%, and 2.5% per SNP, respectively) which improved with additional clinical variables such as sex. CYP2C19*2 also accounted for ∼16% of variation in active metabolite levels (Verma et al., 2020). Interestingly in a controlled genetic cohort in an Amish population CYP2C19*2 accounted for around 12% of genetic variation of platelet reactivity alone compared to the explained variation seen by the 6 SNPs in Lewis et al.’s study (Shuldiner et al., 2009). Further studies utilising an increased cohort size and prospective data will be needed to follow up these interesting findings.

#### 2.10.3 Warfarin

Warfarin remains a widely used anticoagulant, despite the increasing use of direct oral anticoagulants (DOACs), and remains the mainstay of long-term anticoagulation therapy for those with mechanical heart valves, paediatric patients, those patients with severe renal impairment, and for patients in many parts of the developing world. Despite its effectiveness as an oral anticoagulant, warfarin is responsible for a significant bleeding risk of 7.2 events per 100 patient years. Moreover, it is in the top three drugs responsible for hospital admissions, largely due to the narrow therapeutic window and large inter-patient response variability (Pirmohamed, 2018). A concerted effort has been made to improve warfarin safety with development of polygenetic and clinical dosing algorithms.

In 2009, the International Warfarin Pharmacogenetics Consortium (IWPC) developed a pharmacogenomic algorithm, incorporating VKORC1 and CYP2C9 genes, which improved initial dosing of warfarin compared with clinical algorithms and fixed dosing regimens; the improvement was greater at dosing extremes (<21 mg and >49 mg per week) (International Warfarin Pharmacogenetics et al., 2009). Following this, numerous trials have compared pharmacogenomic dosing to clinical algorithms and standard care with mixed results; the three largest trials to date are COAG, EU-PACT, and GIFT. The COAG trial compared a clinical to a pharmacogenomic algorithm and showed no difference between groups for time in the therapeutic international normalised ratio (INR) range in the initial 4 weeks of therapy (Kimmel et al., 2013). However, this trial was limited by including 27% African-American patients but without considering variants that impact warfarin dose requirements and are more common in those of African ancestry, such as CYP2C9*5, *6, *8, *11, and rs12777823 G>A (Drozda et al., 2015; Limdi et al., 2015). Furthermore, there is a general lack of warfarin dosing algorithms for African ancestry individuals (Drozda et al., 2015; Pirmohamed, 2018). Conversely, the EU-PACT trial demonstrated greater time in the therapeutic INR range, fewer incidences of excessive anticoagulation and a shorter time to therapeutic INR for those dosed with a pharmacogenomic algorithm compared with standard dosing (Pirmohamed et al., 2013). Lastly, the GIFT trial, which is the largest and most recent warfarin pharmacogenomics trial, assessed perioperative warfarin dosing and reported a decrease in a composite clinical outcome using the pharmacogenomic dosing algorithm, driven mostly by a reduction in episodes of INR >4 and a borderline significant reduction in major bleeding (Gage et al., 2017). In contrast, a recent retrospective real-world Finnish Biobank study observed the effects of *VKORC1* and *CYP2C9* variants on adverse drug reactions to Warfarin. They did demonstrate sensitive and highly sensitive responders had more INR tests above 3. However, this failed to translate into an increase in risk of bleeding complications (Vuorinen et al., 2021). This effect may be due to considering all major and minor bleeding events, although minor bleeding events have previously been shown not to be associated with genotype (Tomek et al., 2013). Moreover, the study assessed long follow up times when most of the benefit from genetics is accrued early after starting warfarin (Schwarz et al., 2008). Due to these disparities (Table 1), even though cost effectiveness in the positive trials has been demonstrated, polygenic algorithmic-based dosing of warfarin is yet to translate into routine practice (although to a large part, this is because the newer direct oral anticoagulants have taken over from warfarin in the treatment of thromboembolic conditions).

**TABLE 1:** Compares the polygenic risk score trials in Warfarin prescribing.

Author	Year	Patient cohort Size	Patient ethnicity	SNPs/Genotypes included	Primary outcome Measure(s)	Comparison warfarin dosing model	Results
Pirmohamed et al.*	2013	455	Largely White British and Swedish	CYP2C9*2, CYP2C9*3, and VKORC1 (−1639G→A)	The percentage of time in the therapeutic INR range of 2.0–3.0 during first 3 months of therapy	NP	Unadjusted percentage of time with an INR in therapeutic range was 67.4% in the genotype-guided group as compared with 60.3% in the control group- difference of 7% between groups (95% confidence interval, 3.3–10.6; P< 0.001)
Gage et al.*	2017	1650	Largely White American	VKORC1-1639G>A, CYP2C9*2, CYP2C9*3, and CYP4F2 V433M	Composite of adverse events: major bleeding within 30 days, INR of 4 or greater within 30 days, death within 30 days, and symptomatic or asymptomatic VTE confirmed by objective testing within 60 days of arthroplasty	CM	10.8% of genotype group vs. 14.7% of clinical model guided group experienced at least 1 composite endpoint. Absolute risk difference of 3.9% (95% CI, 0.7%–7.2%; *p* = 0.02)
Jorgensen et al.*	2019	212	White European	CYP2C9*2 (rs1799853), CYP2C9*3 (rs1057910) and VKORC1 -1639G→A (rs9923231)	Percentage time in target INR range (2.0–3.0) during the first 3 months of treatment	NP	Mean percentage time in range was 62.74% in the implementation group vs. 55.25% in the combined control and normal clinic data for the region. control group. A difference of 7.49%
Kimmel et al.	2013	1015	White American, Hispanic, African American	CYP2C9*2, CYP2C9*3, and VKORC1	Percentage of time participants spend within the therapeutic INR range (2.0–3.0) during the first 4 weeks of therapy	CM	Overall no difference mean percentage time in therapeutic range- 45.2% in the genotype guided group and 45.4% in the clinically guided group

CM = Clinical/phenotypic model, NP = normal clinical practice, * trials which support genetic testing.

#### 2.10.4 Taxanes

A retrospective analysis of the CALGB 40502 taxane trial using genetic data was performed in 2018 to produce a novel PRS combined with clinical data to improve prediction of progression free survival in patients on taxane-based therapy with advanced breast cancer. The resulting risk score had an AUC of 0.81, compared to 0.64 using clinical covariates alone. The initial phase of the genetic study focussed on those of European ancestry, but the final risk score was validated in those of non-European ancestry. The original trial found no superiority in the trial agent over the current clinical standard, paclitaxel, and so has been of limited clinical benefit in helping clinicians differentiate between the treatment options (Rashkin et al., 2019). Their methodology, however, demonstrates potential for other agents in the future, by predicting which agents will be effective for different patients to optimise outcome whilst minimising harm.

#### 2.10.5 Neuropsychiatric agents

##### 2.10.5.1 Antipsychotics

There has been much candidate gene research and in recent years increasing GWAS analysis investigating both antipsychotic efficacy and antipsychotic ADR prediction with mixed/conflicting results (Zhang and Malhotra, 2018). A significant proportion of patients fail to respond to conventional antipsychotic therapy and are “treatment resistant”. The only evidence-based therapy for treatment resistant schizophrenia is clozapine, which comes with a risk of significant adverse drug reactions and generally takes years of antipsychotic trials before initiation leading to prolonged periods of untreated psychosis and poorer prognosis (Schennach et al., 2012). A PRS developed with a small cohort of schizophrenia patients comparing clozapine takers and clozapine naïve patients demonstrated PRSs were higher in the group that required clozapine therapy (Frank et al., 2015). On the other hand, a larger study using standard psychiatric interviews demonstrated a schizophrenia PRS was no better than clinical factors in determining individualised poor outcomes (Landi et al., 2021). Furthermore, a small Danish study analysing treatment resistant schizophrenia, defined as those on clozapine and those hospitalised whilst on monotherapy, found no significant association between PRS and treatment resistance (Wimberley et al., 2017). One study assessing lurasidone efficacy in patients with chronic schizophrenia utilised two phase III trial cohorts: Meltzer et al. (2011) and Nasrallah et al. (2013). The study captured a mixture of Caucasian and African American patients. They identified a group of risk SNPs that together successfully predicted outcome with treatment for Caucasian patients (Li et al., 2018). Unsurprisingly, this effect was not seen in African American patients given the PRS was developed in a Caucasian population. Similarly, to Frank et al. (2015) work with clozapine, increased PRS scores correlated with increased treatment response with lurasidone.

However, in patients with a first presentation of acute schizophrenia and naive to therapy, the opposite results were demonstrated. Patients with a higher schizophrenia PRS value were associated with poorer treatment response in initial treatment (Zhang et al., 2019).

Meta-analysis of all of the candidate gene studies assessing antipsychotic related weight gain was conducted by Zhang et al. (2016). The lead SNP from the six most promising genetic regions was selected and included in a weighted PRS used in both adult and paediatric cohorts to assess for weight gain at 3 months. The generated PRS showed weak correlations with weight gain with a R^2^ value of 0.056.

Results from PRS antipsychotic effectiveness studies appear conflicting and thus far have been reliant on small scale studies. Nevertheless, the proof of concept ideas studied highlight the potential clinical utility for a personalised medicine approach.

##### 2.10.5.2 Antidepressants

Antidepressants are the first line drug for major depressive disorder and there are >30 drugs on the market currently. Around one third of patients will respond to first line antidepressant therapy with another third requiring an alternative agent and the final third fail to respond to at least two agents (Garcia-Gonzalez et al., 2017). Data from seven antidepressant pharmacogenomic trials were included in the largest scale study to date assessing antidepressant response. However, a PRS for antidepressant efficacy was not significant and the polygenetic liability to MDD or schizophrenia did not influence response to antidepressants. The latter finding suggests there are differences in genetic risk variants between depression susceptibility and treatment efficacy (Garcia-Gonzalez et al., 2017).

A study in a much smaller cohort of patients assessed a MDD PRS and a PRS for neuroticism for association with response to SSRIs in patients with MDD. They too were unable to produce a model which remained significant after correction for multiple testing. However, they did demonstrate a direction of effect where higher PRS for MDD and neuroticism were associated with less favourable response to SSRIs (Ward et al., 2018), which should be followed up in larger samples.

Another study using smaller numbers of genetic variables, that also incorporated clinical factors into the risk scores was able to predict treatment response in patients with a diagnosis of MDD who were randomly allocated to receive either escitalopram, or nortriptyline. In cross-drug specificity analyses, neither the risk score for nortriptyline nor escitalopram predicted remission in the opposing group, suggesting each risk score was drug specific. For escitalopram, the AUC in the model training dataset was 0.8, and it remained a moderate prediction (AUC of 0.77) in the replication cohort. The nortriptyline risk score demonstrated similar AUC results with an AUC of 0.83 in the training cohort and 0.77 in the validation cohort (Iniesta et al., 2018). The sample size for this study was small, however, necessitating replication in larger datasets.

For treatment resistant depression, esketamine nasal spray has been demonstrated to be useful for those at imminent risk of suicide. One study evaluated PRS models to predict remission/improvement in clinical symptoms after 4 weeks of initial therapy in two phase III clinical trial cohorts. Their PRS model for depression did not predict treatment response to esketamine after replication corrections; however, there was a trend towards positive correlation for responder and remission status (Li et al., 2020).

##### 2.10.5.3 Lithium

Lithium is the first line treatment option for patients with bipolar affective disorder; however, treatment response varies between individuals with around 30% being only partially responsive and 25% showing no response (Amare et al., 2018).

There is significant overlap reported between genetic susceptibility of major depression and schizophrenia with bipolar affective disorder; two studies have investigated effects of both a major depression PRS and schizophrenia PRS on prediction of lithium response in bipolar disorder. Furthermore, lithium is not an early treatment for either condition so higher genetic predisposition for either depression or schizophrenia could predict an unfavourable response to lithium. The international consortium on lithium genetics study identified an inverse relationship between treatment response and schizophrenia PRS values. An adjusted OR of 3.46 in favour of a positive treatment outcome with lithium was observed in those within the lowest decile of schizophrenia PRS compared to those in the highest decile (Amare et al., 2018). Similarly, the same inverse relationship between PRS decile and lithium response was observed using the MDD PRS, albeit to a lesser extent (adjusted OR 1.49 comparing the lowest to the highest PRS deciles) (Amare et al., 2021). Interestingly, both studies PRSs explained <1% of genetic variance in the Bipolar disorder patients’ response to lithium (Amare et al., 2018; Amare et al., 2021). This suggests that as PRSs are developed based on larger samples, and potentially based on novel bipolar disorder specific PRS scores, prediction of lithium response could improve significantly in the future.

### 2.11 Clinical trials

Over the past few decades, drug trials for neurodegenerative disease-modifying drugs have largely been unsuccessful with 99.6% of Alzheimer’s disease trials between 2002 and 2012 failing (Cummings et al., 2014). Furthermore, a large proportion of these drug trials failed at the very costly phase three stage. Similar trends have been observed in other neurodegenerative diseases, such as Parkinson’s disease, and one of many suggested reasons for this failure may be imbalance of genetics during trial randomisation (Leonard et al., 2020). This imbalance in clinical trials is supported by growing evidence that different phenotypic presentations of disease are due, in part, to differences in underlying genetic architecture, such as faster progression or predominance of certain symptoms. For instance, in Parkinson’s disease, disease variants in the glucocerebrosidase gene have been linked to poorer survival and increased risk of early dementia (Cilia et al., 2016). This imbalance in clinical trials is encapsulated by the post-hoc analysis of phase III solanezumab trials. Subsets of participants were screened for biomarkers of amyloid status, revealing that 25% of those with mild AD were amyloid negative, which could have led to the lack of expected effect seen in the trial (Chen et al., 2016). Underlying genetic variation is likely to have similar effects to those seen with downstream biomarkers such as amyloid status. One research group has attempted to model the effects of single SNP, multiple SNP, or PRSs on variant distribution after randomisation and they found instances where significant differences after randomisation between investigative arms might confound true therapeutic effects (Leonard et al., 2020). Nevertheless, there is currently limited research on the effects of underlying genetics between treatment arms after randomisation and its impact on clinical trial outcomes. Theoretically, adjusting for underlying differences in genetics could lead to greater trial success and decrease drug development costs.

One such trial adopting a genetic enhanced recruitment process, albeit for monogenetic markers, is the PRIZE trial. The protein, endothelin 1 (ET-1), is encoded by *EDN1*, and is the most potent, longest-lasting vasoconstrictor in humans. Increased production of ET-1 and subsequent binding to the ETA receptor has been demonstrated to promote atherosclerosis (Gupta et al., 2017). The allele, rs9349379-G, has been associated with higher circulating serum ET-1 levels and subsequent coronary microvascular dysfunction without obstructive coronary disease and thus an increased risk of CAD (Ford et al., 2020). The PRIZE study is currently using an enriched genomic recruitment strategy to assess zibotentan, which acts as a potent inhibitor of the ETA receptor, in a patient cohort with microvascular angina. They are aiming to recruit to ensure a rs9349379-G allelic frequency of >50%, which will both investigate the hypothesis that rs9349379-G will act as a novel biomarker associated with treatment response (Morrow et al., 2020) and may also maximise efficacious drug response.

### 2.12 The future of polygenic risk scores

Pharmacogenomics has been the subject of intense research over the last three decades. However, to date, there has been limited implementation into clinical practice for several reasons. The advances in pharmacogenomics led to the first PRS development in 2007 (Wray et al., 2007) and, since then, there has been tremendous growth in samples sizes, GWAS analyses, and PRS-based research; so, what is the future for PRSs?

The lack of ethnic diversity in genetic studies conducted to date is widely documented, with most originating from white, Western, European ancestry populations. From the first 10 years of PRS research, 67% of studies included exclusively European ancestry participants and 19% included only East Asian ancestry participants. Only 3.8% of studies analysed from this time period included cohorts of African, Hispanic, or Indigenous peoples, highlighting the huge disparities in genetic research populations (Duncan et al., 2019). Importantly, it has been demonstrated that the European-ancestry derived PRSs predictive ability is lower in non-European populations (Duncan et al., 2019; Martin et al., 2019). Interestingly, cross-evaluation of data from the United Kingdom and Estonian biobanks, and from a German population, showed that the highest performance of the PRSs with respect to coronary artery disease was highest in their corresponding population dataset, but dropped when applied to different European populations of the same ethnicity (Gola et al., 2020). This highlights the issue that a PRS developed in one population cannot be applied to another population without taking into account population structure (Reisberg et al., 2017).

Further to ethnic disparities, there are also significant sex disparities in PRS research. Women have higher rates and severity of adverse drug effects (Pirmohamed et al., 2004; Melloni et al., 2010; Franconi and Campesi, 2014). Large numbers of medications taken off the market in recent times have been due to disproportionately harmful effects in women (Carey et al., 2017). For many conditions it is generally observed that women feature less in clinical trials than men and this is particularly apparent in CVD trials (Melloni et al., 2010). CVD is the leading cause of death in both men and women and so this disconnect between female representation in clinical trials and clinical practice could be an avenue for future research. For example, in one United Kingdom study assessing hospital admission for ADRs, aspirin was the most common culprit drug (Pirmohamed et al., 2004). Women are more likely to experience both thrombosis and bleeding linked with antiplatelet therapy, compared with men, for cause(s) not fully understood (Wang et al., 2012). Women are also more likely to experience drug-induced QT prolongation (Franconi et al., 2011; Darpo et al., 2014) for reasons we do not fully understand. One potential future use of PRSs could be investigating ADRs specifically in women. However, it is currently unclear what the genetic disparities between the sexes are, and sex chromosomes are often excluded from GWAS analyses (Lambert et al., 2019).

To date, PRSs which have focused on disease risk have outnumbered PRS studies focusing on pharmacogenomic end-points. This is perhaps not surprising given that disease PRSs can compare individuals with and without the disease, and this has been accelerated by the availability of large biobanks such as the United Kingdom biobank. However, the use of disease/no disease stratification is relatively crude and does not take into account disease sub-types. To undertake PRSs on pharmacogenomic end-points (efficacy or safety) is more difficult for many reasons. Pharmacogenomic studies have to date relied on specifically defined end-points, which are often not recorded to the same resolution in large biobanks, and thus sample sizes for pharmacogenomic studies, which have had to rely on field based studies, have been small and may thus lack power. Efficacy end-points used in pharmacogenomic studies require specific tools, such as the Montgomery-Asberg Depression Rating Scale for depression studies, which will not be available in biobanks. For safety end-points, stratification based on severity may be important as some of the single gene variants have been associated with severe, but not the milder, phenotypes (Biswas et al., 2022). Another major issue which needs to be considered in pharmacogenomic PRSs is polypharmacy (i.e. the co-prescription of several drugs, typically more than 5, to the same patient). Polypharmacy increases the risk of drug-drug interactions and may modify the genetic effect. Similarly, the risk of toxicity from a drug may also be modulated by underyling disease, such as renal impairment, which may interact with a genetic risk factor to increase susceptibility (Park et al., 2019). In the medium- to longer-term, improving the resolution of phenotypes within biobanks and standardising data collection tools with core outcomes is going to be essential in order to upscale work on pharmacogenomic PRSs.

There are many social barriers to the future utility of PRSs but, here, we briefly cover the main issues. It is unclear currently the long-term psychological effects of genetic risk knowledge on patients and, furthermore, legislation around information governance with this data will have to evolve to include access, identification, and risk profiling issues. Physician and public education around PRSs would be required to maximise clinical utility and this could lead to either under or over treating patients. For the public, this may lead to perceptions of clinicians withholding treatment (Torkamani et al., 2018).

As PRS research and clinical utility increases, disparities in statistical assessment and genetic risk score design between studies will require standardisation to allow comparisons. For example, in primary and secondary prevention of ischaemic heart disease, Rincon et al. (2019) developed their PRS by summation of the number of risk alleles across genetic variants and then dividing into tertiles. Morieri et al. (2018) used similar methodology, but weighted the effect alleles based on literature risk effects and then standardised their risk score. Mega et al. (2015), however, scored their PRS with the sum of the number of risk alleles for each SNP weighted by the log of the odds ratio reported with the SNP in the original literature. Vaara et al. (2016) used a further altered methodology by summation of the number of weighted risk alleles and then dividing by the total number of SNPs included in each of their respective models. Small variations such as this can have large downstream effects on the overall outcome and prediction of the model. Moreover, different analysis methods focus on making comparisons between different subgroups of patients, such as tertiles (Morieri et al., 2018; Rincon et al., 2019), quintiles (Mega et al., 2015) or analysing the risk score as a whole (Vaara et al., 2016). This variation is echoed in other fields, such as breast cancer, where deciles and extreme upper and lower one percentiles are analysed (Mavaddat et al., 2015; Khera et al., 2018). With ever increasing pressure on healthcare systems, rationing of therapy offered to those who receive benefit in a more personalised way, and using targeted screening as opposed to blanket screening with novel PRSs, could help improve efficiency, as outlined earlier. Costs of whole genome sequencing have rapidly declined, moving from estimates of US$10 million in 2007 to below US$1,500 in late 2015, and are falling further as investment increases (NIH National Human Genome Research Institute, 2016; Payne et al., 2018). Some pilot data on limited numbers of participants has suggested that, as costs reduce, an increased diagnostic yield from genetics and reducing target therapy could indeed become cost effective in clinical practice with obvious differences in diagnostic yield depending on disease process (Sagoo et al., 2017). Whole exome sequencing has already been demonstrated to be more cost effective than traditional diagnostics in paediatric populations with muscle disorders (Schofield et al., 2017), as well as in paediatric monogenetic disorders, particularly when the test is conducted early (Stark et al., 2017). However, mixed success of cost-effective models in adults, such as genetic testing to guide ibrutinib therapy, has demonstrated improved health outcomes but at a significant cost. Unless treatment or diagnostic costs are reduced, this is unlikely to be an acceptable price in current medical practice (Buchanan et al., 2017). Similarly, targeted lung cancer therapy was found to not be cost effective (Doble et al., 2017); however, in dilated cardiomyopathy, family testing before symptoms and guided screening of those individuals is highly likely to be cost effective (Catchpool et al., 2019). Logic would dictate that early genetic testing to predict future disease risk and guide future therapies could be the most cost-effective test in healthcare. However, decisions around who will pay and manage such initiatives will certainly play a role in the future, and currently, healthcare systems still focus on treating rather than preventing diseases.

**FIGURE 1:**
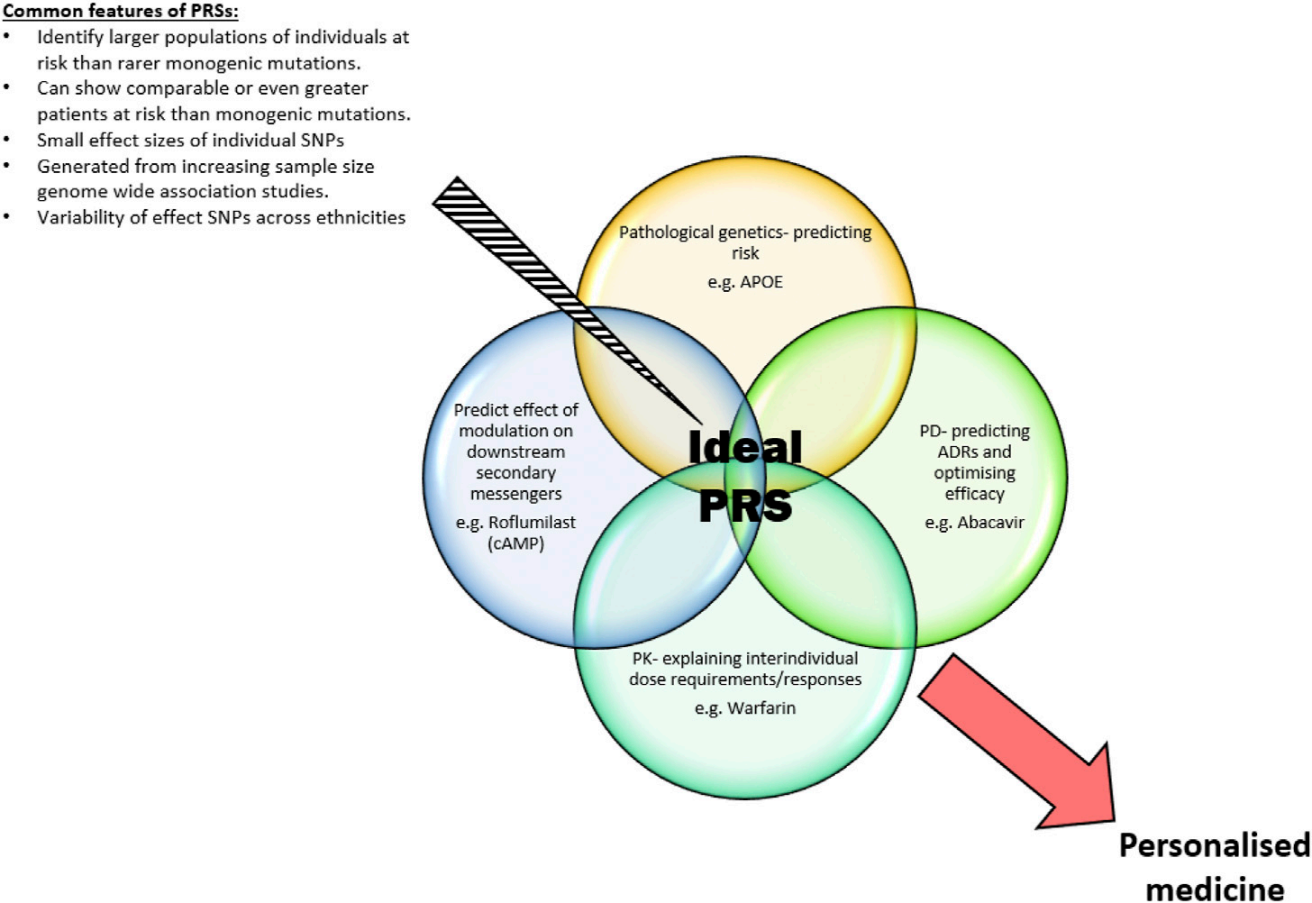
Common features of PRSs and the ideal individualised areas an optimal PRS identifies to produce a personalised medicine approach to future healthcare.

As we learn increasing amounts about the role of genetics, disease development models will have increasing predictability and clinical utility. We are able to detect SNPs with smaller and smaller effect sizes due to larger sample sizes in clinical research and subsequently the number of SNPs reaching genome wide significance continues to rise (Figure 1). Khera et al. (2018) used PRS methodology to identify larger fractions of a population with comparable, if not increased disease risk compared to known monogenic risk variants. This increased detection has further impacted genetic prediction models, demonstrated by a gradual increase in the AUCs for T2DM, breast cancer, coronary heart disease and prostate cancer prediction models from 2007 to 2013 (Krier et al., 2016). The incremental improvements to PRSs over time have also been exemplified in CAD. Importantly, this increase in risk prediction in one PRS crossed the clinically significant 6% threshold from the American College of Cardiology/American Heart Association pooled cohort equations atherosclerotic cardiovascular disease estimator that assesses the value of new biomarkers in addition to current models for making primary prevention predictions in IHD (Morieri et al., 2018). As research increases and more patient genetic data becomes available, the strength of genetic risk models should continue to increase, hopefully increasing clinical utility. At the date of writing, there are currently 22 clinical trials in progress involving PRSs across a variety of pathologies including breast cancer and CVD (Supplementary Material). Further to this, the United Kingdom government’s Department of Health and Social Care green paper on disease prevention has made genetic information a priority of the United Kingdom healthcare system research with the ambition to embed genetic information into routine healthcare practice (UK Department of Health and Social Care, 2019). The ambitious United Kingdom plans are further outlined in the new National Genomic Healthcare Strategy (Department of Health and Social Care, 2020) and are reflected in other healthcare systems globally, such as the CDC considering PRS utilisation in the United States (Khoury, 2019).

With the increasing availability of sequencing data, an important issue which needs further work is how to integrate rare variants together with common variants in order to determine whether this improves predictability. Studies evaluating this aspect are now beginning to appear. Lali et al. (2021) were able to develop a rare variant genetic risk score which was able to identify 1.5% of people with a risk of early for coronary artery disease even when adjusting for Mendelian genes, clinical risk factors and common genetic variants. A recent study showed that PRSs based on common genetic variants for obesity, and the risk of severe obesity and early bariatric surgery, was enhanced by incorporating rare variants associated with significant gene expression changes (which they termed expression outliers), highlighting the potential utility of combining common and rare genetic variants in PRSs (Smail et al., 2022).

**FIGURE 2:**
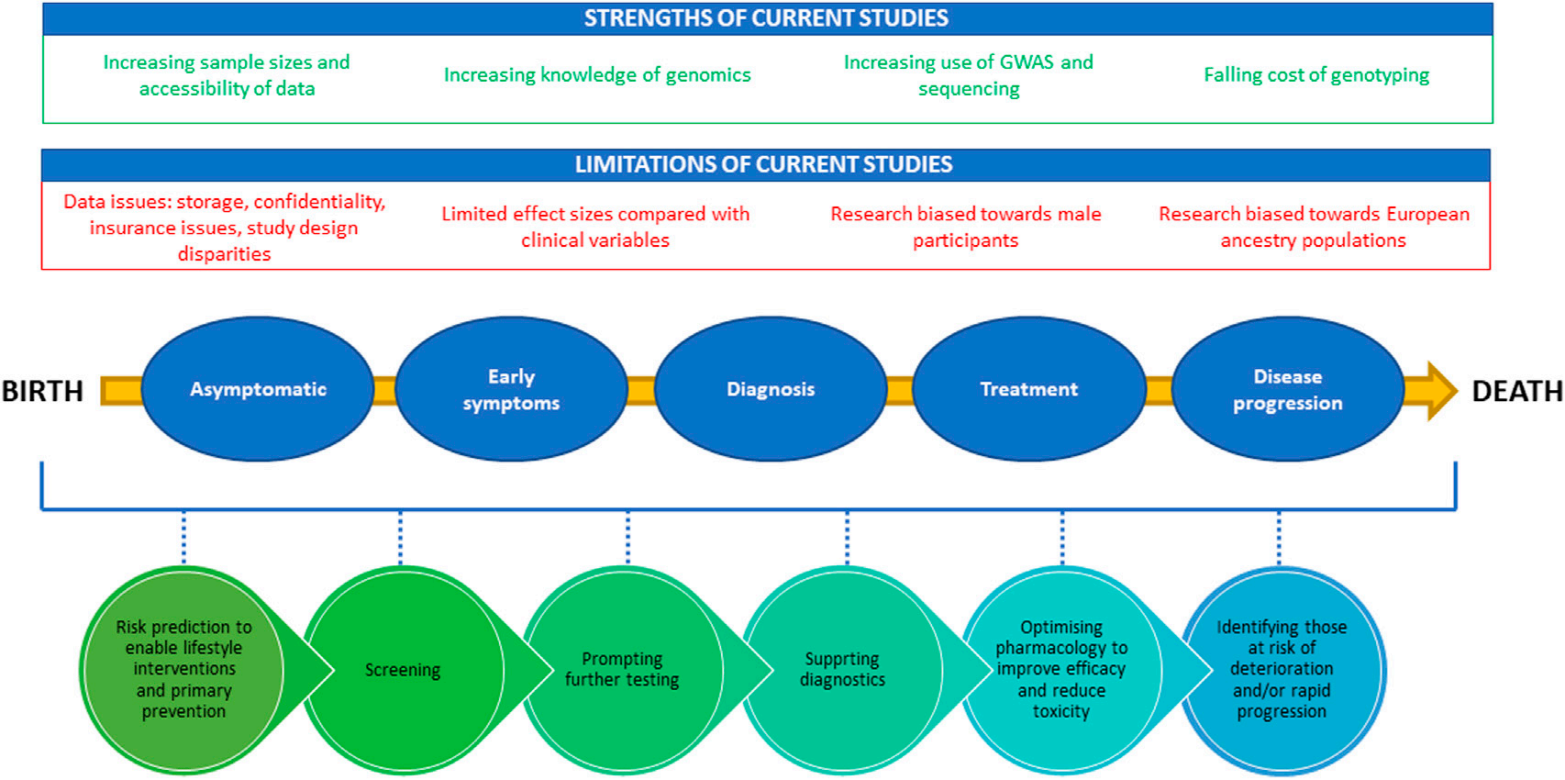
A diagram demonstrating positive and negative factors influencing PRS development and the potential clinical utility of PRSs from birth to death. The strengths of the current studies are highlighted in green at the top of the figure, while limitations are shown in red. The bottom half of the figure provides the potential timepoints for utilization of PRSs through the life cycle from birth to death.

Large GWAS and sequencing studies and the development of PRSs can also provide insight into how different biological pathways interlink and identify potential key genes and downstream metabolites to develop novel drug targets and therapies. GWAS studies have already confirmed or contributed to identification of novel therapeutic targets such as SLC30A8 for T2DM and IL-23 for Crohn’s disease (Nabirotchkin et al., 2020). A recent novel approach to drug targeting, termed ‘*pharmagenic enrichment score (PES)’*, aims to increase the clinical utility of PRSs in complex diseases. The PES approach combines known biological pathways with individuals’ genetic risk for a given disease process with drugs known to act on these pathways to provide an individualised therapeutic strategy to best manage that individual patient’s risk. This strategy also provides the potential to repurpose drugs not commonly used in the disease but known to act on the associated pathway(s) (Reay et al., 2020). It has been estimated that selecting genetically supported drug targets in drug development could double success rates in clinical development (Nelson et al., 2015). It seems that PRSs have the potential to aid target discovery and patient stratification (Figure 2).

The complex issue of how to implement PRS into clinical practice has not been adequately investigated. Implementation of novel products or tests into healthcare is complex and requires multidisciplinary expertise that is often not utilised in the initial discovery studies. Researchers in the PRS field should also bear in mind that implementation of pharmacogenomic variants, even when the effect size is much greater than that seen with PRSs, has been limited despite efforts over decades. The difficulties in implementation of PRSs is going to be further compounded by the fact that PRSs are much more complex comprising large, and differing, numbers of variants, as shown in this review, when compared with pharmacogenomic tests which consist of single/few variants.

Direct to-consumer (DTC) genetics has also expanded rapidly, providing the public with access to individual genetic profiles and interpretation of common genetic variants. Since 2015, a group of academic geneticists have developed an online open access genetics platform which utilises this DTC data to produce PRS scores for various health traits and diseases. The platform touches on uncharted territory at the forefront of current debate about PRS utilisation in clinical practice, including how best to present genetic data to the public and what the lasting effects of knowing one’s genetic profile might be (Folkersen et al., 2020). Growth of other online public platforms, such as openSNP, SNPedia/Promethase and The Personal Genome Project, allow customers to publish their DTC results for research purposes, data aggregation and sharing of scientific information (Greshake et al., 2014).

## 3 Conclusion

Since the first polygenic risk scores were developed, research in this area has progressed at pace. PRSs have been shown to have some potential in disease risk identification, drug targeting and stratified medicine across a range of therapeutic areas including oncology, cardiovascular and psychiatry. Nevertheless, further evidence of clinical utility is required to spark translation and implementation into routine practice. As costs and other barriers impinging genetic testing are navigated, and multi-ethnic sample sizes for development and validation of PRS grow, the future of polygenic risk scores looks cautiously optimistic.
